# Supplementary material for: A Comparative Analysis of 5-Azacytidine- and Zebularine-Induced DNA Demethylation
Source: G3 (Bethesda). 2016 Jul 5;6(9):2773–80. doi: 10.1534/g3.116.030262 (PMC5015934; doi:10.1534/g3.116.030262)
Supplement: Supplemental Material [file supp_6_9_2773__index.html]

A Comparative Analysis of 5-Azacytidine- and Zebularine-Induced DNA Demethylation — Supplemental Material 

# A Comparative Analysis of 5-Azacytidine- and Zebularine-Induced DNA Demethylation

## Supplemental Material for Griffin, Niederhuth, and Schmitz, 2016

**Files in this Data Supplement:**

- Figure S1 - DNA methylation across all chromosomes is decreased when treated with AZA and ZEB in a concentration-dependent fashion. (.pdf, 1 MB)
- Figure S2 - AZA and ZEB induce a concentration-dependent decrease in DNA methylation in all types of genetic elements. (.pdf, 460 KB)
- Figure S3 - Pairwise comparison of highly methylated 100 bp windows between ZEB-treated and control seedlings. (.pdf, 129 KB)
- Table S1 - Reads, Coverage, and non-conversion. (.xlsx, 9 KB)
- Table S2 - Genome-wide methylation level-Deeply sequenced and FASTmC. (.xlsx, 9 KB)
- Table S3 - Linear regression on Figure 1A (Concentration v. Methylation level). (.xlsx, 9 KB)
- Table S4 - Differential expression of AZA-treated seedlings in different genetic elements. (.xlsx, 10 KB)
- Table S5 - Top 110 upregulated genes. (.xlsx, 12 KB)
