# Supplementary material for: A Comparative Analysis of 5-Azacytidine- and Zebularine-Induced DNA Demethylation
Source: G3 (Bethesda). 2016 Jul 5;6(9):2773–80. doi: 10.1534/g3.116.030262 (PMC5015934; doi:10.1534/g3.116.030262)
Supplement: Supplemental Material [file supp_g3.116.030262_FigureS1.pdf]

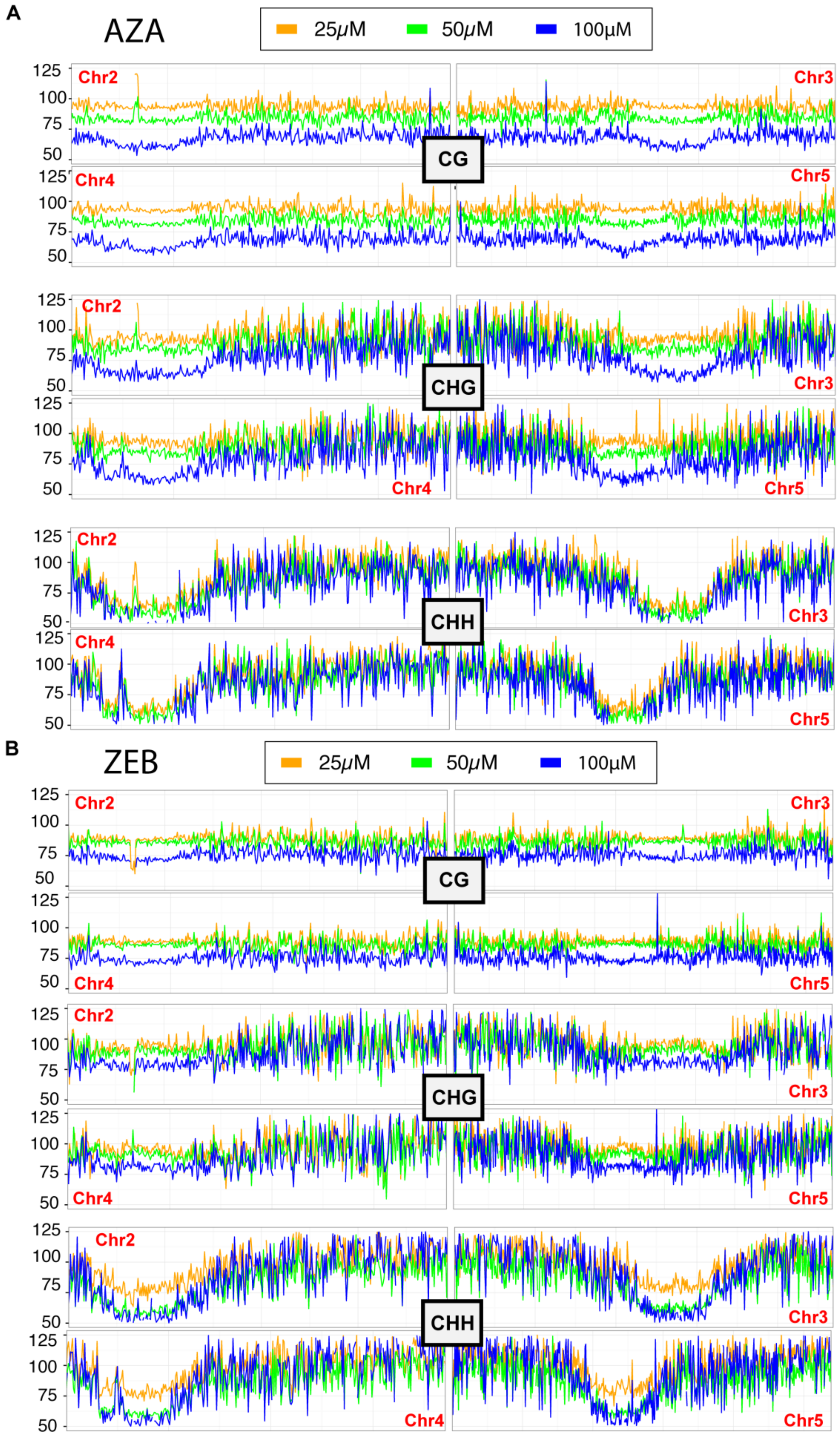

A
AZA
25μM
50μM
100μM
CG
CHG
CHH
Chr2
Chr3
Chr4
Chr5
125
100
75
50
B
ZEB

Figure S1 | DNA methylation across all chromosomes is decreased when treated with AZA and ZEB in a concentration-dependent fashion

A-B. Relative methylation level shown across chromosomes 2-4 (chromosome 1 shown in Figure 2B) for AZA-treated (S1A) and ZEB-treated (S1B) seedlings.
