# Supplementary material for: A Comparative Analysis of 5-Azacytidine- and Zebularine-Induced DNA Demethylation
Source: G3 (Bethesda). 2016 Jul 5;6(9):2773–80. doi: 10.1534/g3.116.030262 (PMC5015934; doi:10.1534/g3.116.030262)
Supplement: Supplemental Material [file supp_g3.116.030262_FigureS2.pdf]

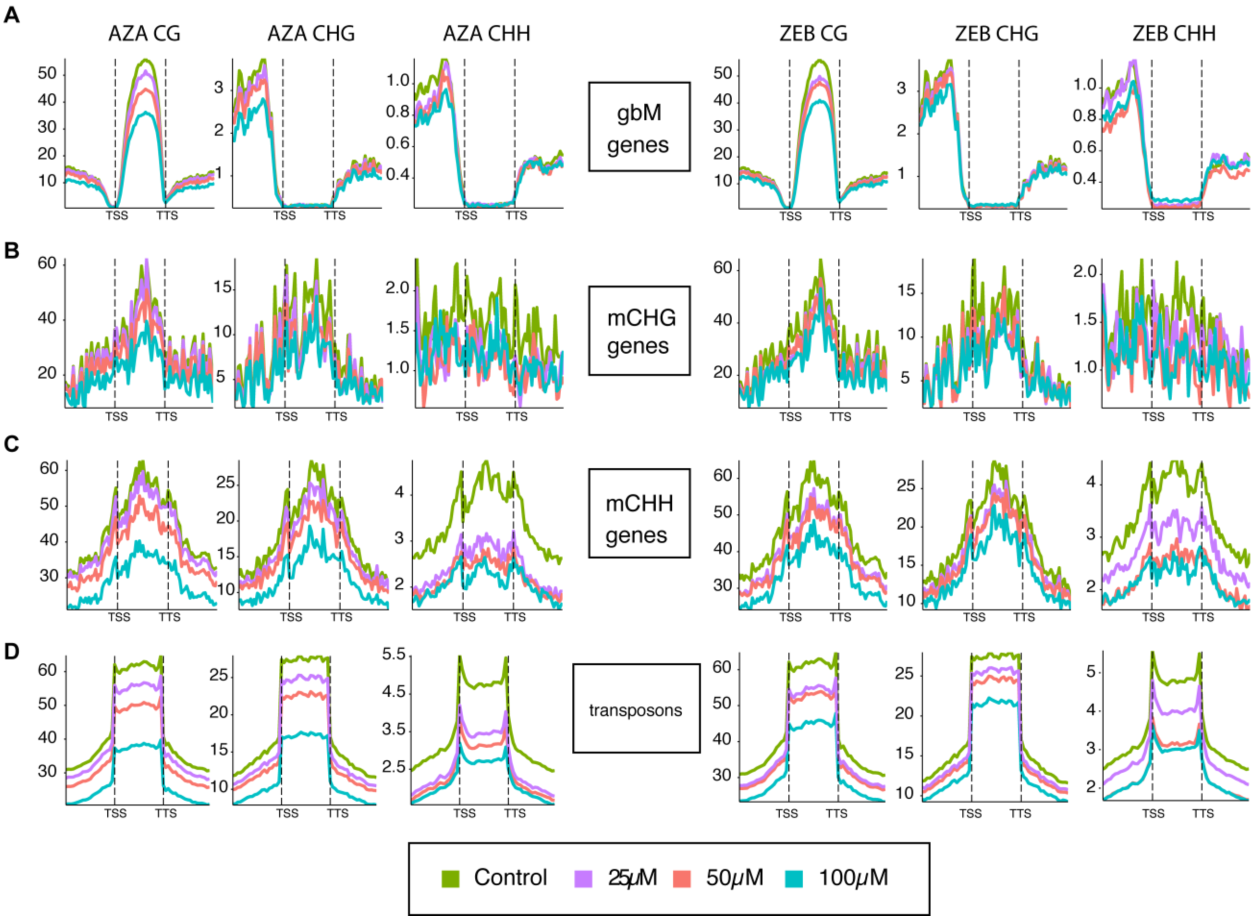

Figure S2 | AZA and ZEB induce a concentration-dependent decrease in DNA methylation in all types of genetic elements

A-D. DNA methylation maps across four different categories of methylated genetic elements: gbM genes (A), CHG-enriched (mCHG) genes (B), CHH-enriched (mCHH) genes (C), transposons (D). The colors (top) represent the different treatment concentrations. The demethylating agent and sequence context is given above each vertical column of graphs.
