# Supplementary material for: A Comparative Analysis of 5-Azacytidine- and Zebularine-Induced DNA Demethylation
Source: G3 (Bethesda). 2016 Jul 5;6(9):2773–80. doi: 10.1534/g3.116.030262 (PMC5015934; doi:10.1534/g3.116.030262)
Supplement: Supplemental Material [file supp_g3.116.030262_FigureS3.pdf]

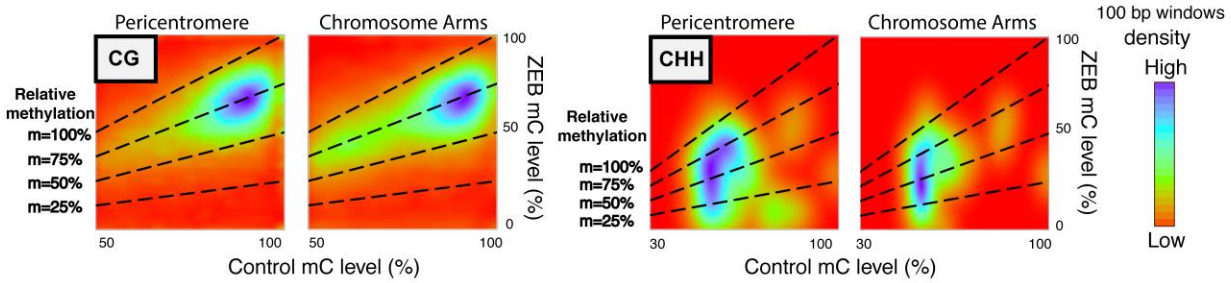

Figure S3 | Pairwise comparison of highly methylated 100 bp windows between ZEB-treated and control seedlings

A pairwise comparison of methylation level in untreated seedlings and the 100 µM ZEB-treated seedlings for highly methylated 100 bp windows in both the pericentromere and the chromosome arms (as defined in Figure 2A). CG and CHH contexts of DNA methylation are shown. A highly methylated window was defined as having ≥50% methylation in the control sample for CG and ≥30% methylation in the control sample for CHH. ZEB-treated seedling methylation level is on the y-axis and control methylation level is on the x-axis. The color spectrum—ranging from red (low) to purple (high)— illustrates the density of points at a coordinate. The slopes (m) of the dashed lines represent the following relative methylation levels: 100% (treated and control methylation level are the same), 75%, 50% (treated methylation level is half of the control), and 25%.
